# An Assessment of Hazards Caused by Electromagnetic Interaction on Humans Present near Short-Wave Physiotherapeutic Devices of Various Types Including Hazards for Users of Electronic Active Implantable Medical Devices (AIMD)

**DOI:** 10.1155/2013/150143

**Published:** 2013-09-08

**Authors:** Jolanta Karpowicz, Krzysztof Gryz

**Affiliations:** Laboratory of Electromagnetic Hazards, Central Institute for Labour Protection-National Research Institute (CIOP-PIB), Ulica Czerniakowska 16, 00-701 Warszawa, Poland

## Abstract

Leakage of electromagnetic fields (EMF) from short-wave radiofrequency physiotherapeutic diathermies (SWDs) may cause health and safety hazards affecting unintentionally exposed workers (W) or general public (GP) members (assisting patient exposed during treatment or presenting there for other reasons). Increasing use of electronic active implantable medical devices (AIMDs), by patients, attendants, and workers, needs attention because dysfunctions of these devices may be caused by electromagnetic interactions. EMF emitted by 12 SWDs (with capacitive or inductive applicators) were assessed following international guidelines on protection against EMF exposure (International Commission on Nonionizing Radiation Protection for GP and W, new European directive 2013/35/EU for W, European Recommendation for GP, and European Standard EN 50527-1 for AIMD users). Direct EMF hazards for humans near inductive applicators were identified at a distance not exceeding 45 cm for W or 62 cm for GP, but for AIMD users up to 90 cm (twice longer than that for W and 50% longer than that for GP because EMF is pulsed modulated). Near capacitive applicators emitting continuous wave, the corresponding distances were: 120 cm for W or 150 cm for both—GP or AIMD users. This assessment does not cover patients who undergo SWD treatment (but it is usually recommended for AIMD users to be careful with EMF treatment).

## 1. Introduction

Electromagnetic fields (EMF) directly affecting living organisms cause an electric field to be induced in the exposed body. In the case of radiofrequency fields with a frequency exceeding 10 MHz, the dominating direct exposure effect in human body is a thermal effect, which may cause an increase in tissue temperature [1]. This effect may be used intentionally for physiotherapeutic treatment, including pain reduction therapy [2]. It is also recognised as the main direct effect of exposure to high-frequency EMF, which creates health hazards because of possible thermal damage to tissues in the case of intentional or unintentional overexposure [1]. It may also be among the components of health hazards associated with long-term (many years) unintentional side exposure to radiofrequency (RF) EMF (e.g., at the workplace or because of the use of wireless communication facilities), summarised by the IARC 2B classification of possible carcinogenic exposure for humans and by reports regarding other possible health dysfunctions increasing among exposed populations [3–5].

EMF affecting an electrically conductive object, for example, a metallic one, also directly induces an electric voltage in it. When such an object includes electronic components, the EMF interactions may create dysfunctions in the electronic devices [6]. When humans are affected by the results of the function of the electronic devices, any uncontrolled interaction may create health or safety hazards. Highly sensitive relations between humans and electronic devices are established for example where any electronic active implantable medical device (AIMD) is supporting a vital function of the human body, such as in the use of cardiac peacemakers, implantable cardioverter defibrillators, cochlear implants, implantable neurostimulators, implantable infusion pumps or another type of implantable medical devices [7]. Various possible dysfunctions of AIMD reported in the research papers presenting observations of the results of exposure to RF EMF of various frequencies include, for example, the destruction of cochlear implants when an electrosurgical unit is used for surgery on the user (usually related with exposure to RF EMF of 0.3–2 MHz frequency); the recipient of a cochlear implant hearing distorted sound when passing near or through EAS (electronic article surveillance—emitting RF EMF from various frequency ranges, mostly from 20 kHz up to 20 MHz) and metal detection gates (emitting kHz or even lower frequency EMF); an overdose of insulin from infusion pumps exposed to EMF from mobile phones or RFID (radio frequency identification devices, usually emitting RF EMF of 0.8–2.4 GHz); changes in the operating rate of cardiac implants; and dysfunctions of neurostimulators [8–11].

Therapeutic devices emitting a RF EMF at a frequency of 27 MHz (recognised as short wave diathermia devices-SWDs) are widely used in physiotherapeutic medical centres, for treatments such as pain reduction. SWD may function equipped with capacitive applicators or inductive applicator, supplied electrically by a continuous wave (CW) radiofrequency signal or by a pulsed modulated one (PM). The output power of SWD used to treat patients is regulated in the range from several watts to several hundreds of watts. The modulation of the signal supplying applicator may be also regulated. SWDs with capacitive applicators are usually CW supplied, but SWDs with inductive applicator are frequently PM supplied, sometimes it is regulated by the operator—CW or PM emission from applicators. During the patients' treatment, as a result of EMF leakage from the applicators and supplying RF cables, not only the patient undergoing SWD treatment is exposed to EMF (intentionally), but also humans and objects nearby, exposed unintentionally [4, 6, 12, 13]. Through the use of SWD, health care workers (SWD operators) may be exposed to EMF produced by the active device while changing settings of the treatment parameters and changing the location of the applicator around the patient's body. In the course of regular treatment, usually operators may stay away from the active applicator (even in the distance of a several meters) where exposure level is significantly reduced in the result of distance from the EMF source. The distance between active EMF source and all workers employed in the treatment centre (SWD operators, nurses, administrative personnel, masseurs, etc.) depends on the spatial organisation of the treatment room and other adjacent rooms. This distance may be even shorter than 1 meter, when, for example, the administrative work space is situated just at the opposite side of the light weight nonconductive wall of the treatment box (e.g., made from the plaster-board modules, which do not screen EMF). Sometimes also assisting to disabled patients may be needed and any health care worker or patient's attendant (e.g., their relative) has to stay near patient and active SWD over the duration of the treatment. The space which is accessible for patients waiting for any treatment, patients undergoing other treatments, or workers doing such treatments, for example massage, may also be located in the vicinity of active SWD. In the result, various groups of humans, health care workers operating SWD (SWD operators), other workers present in the treatment centre, any patients present near active SWDs or patient's attendants (usually relatives), may be subjected unintentional exposure to RF EMF from active SWD being in the small distance from this devices, over the whole duration of treatment of an order of 10–20 minutes or just passing by and being exposed over of fraction of minute only.

There is usually a warning note in SWD manuals that the application of RF EMF to patients who use AIMD is not recommended or has to be consulted with responsible medical doctor. However, AIMD users may also be among other patients who are present near SWD because of another treatment or waiting for treatment, as well as attendants who assist patients and health care workers employed in the medical centre, who are SWD operators or not. The safety data regarding their possible sensitivity to the AIMD dysfunctions caused by EMF exposure near SWD are scarce, but in the literature it is possible to find already mentioned reports on the AIMD dysfunctions caused by RF EMF exposure of various frequencies (both, lower, and higher than 27 MHz emitted by SWD, as well as of electric, E-field, or magnetic, H-field, dominating component). Such reports indicate that various AIMD functions may also be affected by RF EMF emitted by SWD and such potential hazards should be covered by assessment of safety near SWD. Direct effects of EMF exposure should be also assessed because of the requirements of labour law or international guidelines. Scarce data regarding EMF from SWD which are available in the research papers have got many limitations, such as the assessment focused on direct effects of SWD operators EMF exposure only and only from the old models of SWD and nonsystematics methodology of reported measurements without attention to differences between CW and PM signals measurements and assessment [4, 12–15]. 

The New European Directive 2013/35/EU on the protection of workers against health and safety hazards caused by EMF exposure stresses the need to assess any direct and indirect hazards from electromagnetic interaction on workers, with special attention to the users of AIMD [16].

- Direct effects are recognised as interaction with the human body, assessed by EMF at the workplace and induced in the body, with respect to the thermal effects of exposure (investigated by E- and H-field measurements near SWD and assessed in accordance with the exposure limits called action levels).
- Indirect effects are recognised as interaction with objects, for example, interference with AIMD, including cardiac pacemakers and other implants or contact currents in humans who are in contact with objects exposed to EMF).

Proper identification of the location of the area where possible EMF hazards may exist near SWD of various types is necessary for the safety of the people present in physiotherapeutic centres. The nature of indirect EMF hazards caused near SWD by unintentional RF EMF exposure for AIMD users who are not workers is the same as for workers, and safety of both groups of AIMD users may be assessed following unified rules. 

Direct EMF hazards consist of thermal effects of exposure and are assessed following different rules provided for exposure to the general public and workers. In both assessments, the procedure of the assessment is based on international guidelines provided by the International Commission on Nonionizing Radiation Protection [1]. This assessment does not regard patients who undergo SWD treatment. International guidelines and legislation on workers and general public EMF exposure advise also to limit the level of EMF exposure much more than exposure received by patients undergoing SWD treatment.

The aim of the work was to study the parameters of RF EMF near SWD therapeutic devices with respect to requirements for protection against the direct and indirect effects of exposure. The need for studies that focused on EMF from these medical devices was identified following international advice. Medical devices intentionally emitting EMF are identified by European Standard EN 50499 among devices that, if used in the workplace, require an assessment of occupational EMF hazards [17]. Short-wave diathermy is indicated as a source of possible high EMF exposure in a review by Hansson Mild et al. [6]. The new important aspect of presented study was to distinguish between assessment based on limits provided to protect against direct hazards for workers and general public (with regard to the time-averaged RF EMF exposure) and limits provided to protect against indirect hazards for AIMD users (with regard to the exposure not averaged in time). In consequences it was also needed to follow both types of limits in the analysis of measurement results (by performing detailed analysis of RF EMF exposure pattern in the time domain and analysis of the consequences of it for the “behaviour” of EMF measurement devices and their indications) and to assess RF EMF hazards with respect to the 3 categories of exposed humans: workers (W), general public (GP) members, and AIMD users.

The scale of the public health attention that should be related to the investigated problem may be exemplified by the following. Based on public info on the health care services, in Poland (38,000,000 population) it was estimated that 5–10 thousand SWDs are in use in health care centres, where at least a half of that number of workers are operating these devices. Implanted medical devices are spreading among the population, who may be present near SWD because of employment there or due to visiting the health care centre for any treatment or any other reason. The number of AIMD users is growing each year. The most popular today are implants used by diabetics and people with cardiac dysfunctions. Following the World Health Organization (WHO) data, 10–15% of diabetics need insulin, and 40% of them may use insulin pumps (in Poland it is a population on the scale of 100,000 patients). In Poland, over 30,000 cardiac implants are implanted every year. The use of cochlear implants is also rising steadily; the current estimation is that over 2,000 patients already have cochlear implants in Poland. The use of AIMD in other European countries is at least on a similar scale in the proportion of the population and rising.

## 2. Materials and Methods

Following these recommendations, the presented study was performed near 12 radiofrequency physiotherapeutic short-wave diathermies (SWDs). The exposure assessment was carried out with respect to requirements regarding the health and safety of workers (including SWD operators) and members of the general public (e.g., a patient who does not undergo radiofrequency treatment or attendants assisting the disabled patient) against the harmful effects of EMF exposure, as long as with requirements for the protection of AIMD against dysfunctions caused by EMF interactions, that is, to perform an EMF assessment regarding both direct and indirect electromagnetic hazards caused by physiotherapeutic SWD [1, 7, 16, 18].

The study performed near SWD concerned the distribution of electric and magnetic fields (E-field expressed in volts per meter (V/m) and H-field expressed in amperes per metre (A/m)) in the vicinity of applicators. Electric fields induced in the body and capacitive electric currents caused by EMF interaction in workers operating these devices were not assessed. 

**Figure 1:**
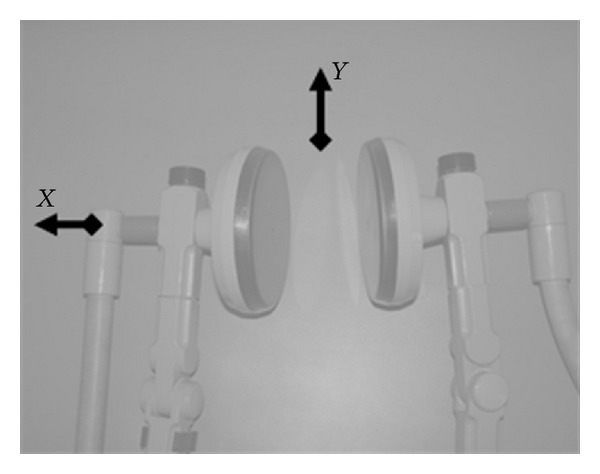
The scheme of measurements performed near SWD applicators.

Measurements of the root-mean-square (RMS) values (i.e., time-averaged exposure metrix) of H-field and E-field strength were performed using a Narda EMR 300 meter with an H-field probe (0.02–16 A/m; 0.3–30 MHz) and an E-field probe (1–800 V/m; 0.1–3,000 MHz). Measurements were performed with a plastic container filled with 1.5 liter of 1% saline used as a patient's phantom for SWD. The reported measurements were performed along orthogonal distances from the cover of the applicators (Figure 1). 

In the case of assessing direct EMF hazards, all measurement results were analysed in order to obtain E-field and H-field values averaged over any six-minute period, that is, time averaged RMS value according to the requirements provided by ICNIRP, European EMF Directive 2013/35/EU, and Council Recommendation 1999/519/EC regarding hazards caused by thermal effects [1, 16, 18]. Indirect EMF hazards were assessed according to the opinion given by European Standard No. EN50527-1:2010 that the electromagnetic immunity of AIMDs depends on the EMF values nonaveraged over time [7]. It means that, in the case of PM EMF, the rules of an assessment of direct and indirect effects are different. And also the metrological properties of used EMF measurement devices, calibrated in the sinusoidal time-varying EMF, are different in the case of measurement of continues wave (CW) EMF and pulse modulated (PM) EMF.

The international standard IEC EN 60601-1-2, concerning general safety requirements for medical life-supporting devices, does not cover EMF emitted by the investigated physiotherapeutic equipment (the immunity of medical devices is tested in radiated EMF with the frequency range 80–2,500 MHz, at the 10 V/m level of E-field, which covers common environmental exposures to EMF emitted by radio and television broadcasting and wireless communication systems facilities) [19]. 

AIMDs intended for the European Union market have to be compliant with the European Standard EN50527-1, which requires that when EMF does not exceed the reference level for the general public given by ICNIRP requirements and Council Recommendation regarding EMF of 0–300 GHz frequency, then AIMD dysfunctions caused by EMF should not be expected by users [1, 7, 18]. This means that, at 27 MHz, the E-field exposure of up to 28 V/m level (28 V/m RMS value in the case of the sinusoidal CW field or peak in-time RMS in the case of PM field) and H-field exposure of up to 0.073 A/m level can be considered as safe for all AIMD users in European countries. In the case of exposure exceeding such limits some AIMD may also function properly, but it needs individual assessment.

Workers' protection against the thermal effects of EMF exposure is ensured by the European directive 2013/35/EU and ICNIRP guidelines when the 6-minute averaged RMS value of E-field does not exceed 61 V/m and of H-field does not exceed 0.16 A/m at 27 MHz [1, 16]. General public exposure limitations, expressed in a 6-minute averaged RMS value provided by ICNIRP guidelines and EU Recommendation, are 28 V/m and 0.073 A/m [1, 18].

**Figure 2:**
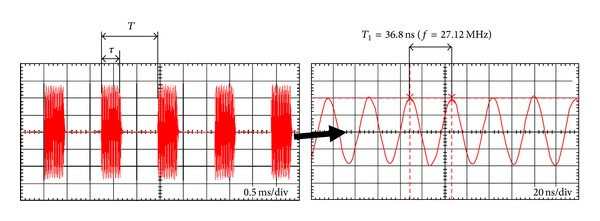
Oscillogram of pulse modulated EMF emitted by SWD-E (duty cycle *D* = *τ*/*T* = 0.30).

Following these rules, from all the results of RMS value E-and H-field measurements, the peak in-time RMS values of pulsed (PM) or continuous wave (CW) emissions is derived, taking into account parameters of modulation identified by oscilloscopic observations and metrological properties of the used measurement devices. The correction factor applied for PM EMF in the assessment of AIMD hazards is expressed by the formula

(1) $$\begin{array}{c} K=\frac{1}{D^{1/2}}, \end{array}$$

where duty factor, *D*, expressed the main parameter of PM—the duration of pulses (*τ*) emitted by SWD divided by the pulse repetition time (*T*), (Figure 2).

For the CW exposures correction factor *K* is “by definition” equal to one and therefore can be omitted.

## 3. Results and Discussion

Investigations were performed near the following typical physiotherapeutic devices:

- short-wave diathermia equipped with a capacitive applicator (SWD-E), emitting a EMF continuous sinusoidal wave (CW) of 27 MHz frequency and an output power of up to 350 W;
- short-wave diathermia equipped with an inductive applicator (SWD-H), emitting a pulse modulated (PM) EMF of 27 MHz frequency, with a duty cycle *D* = 0.001625–0.45, an output power of up to 1000 W peak, and averaged output power up to 90 W.

The main parameters of the investigated SWDs are given in Table 1. The settings of SWDs are reported following the indications at the control panel of devices, but duty cycles were also controlled during measurements by the oscilloscopic observations.

**Figure 3:**
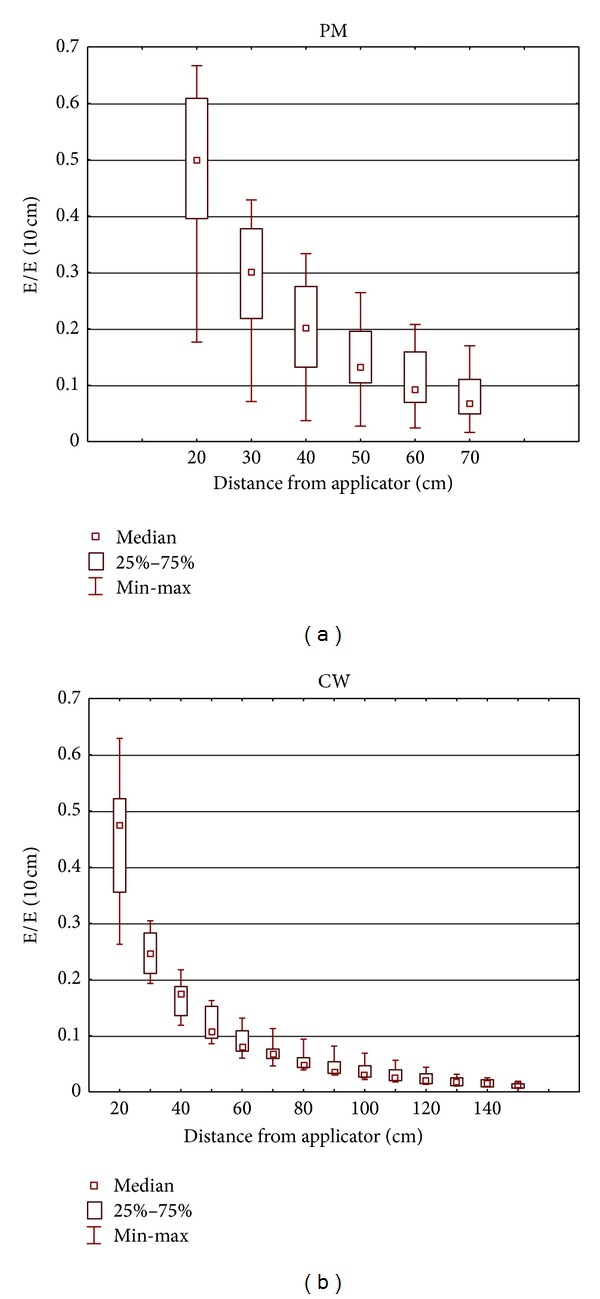
The normalised distribution of the E-field (to the E-field measured at a distance of 10 cm from the cover of applicator) near the applicators. The statistical parameters of all measurement results: (a) SWD-H emitting PM EMF (no. = 9); (b) SWD-E emitting CW EMF (no. = 3).

**Figure 4:**
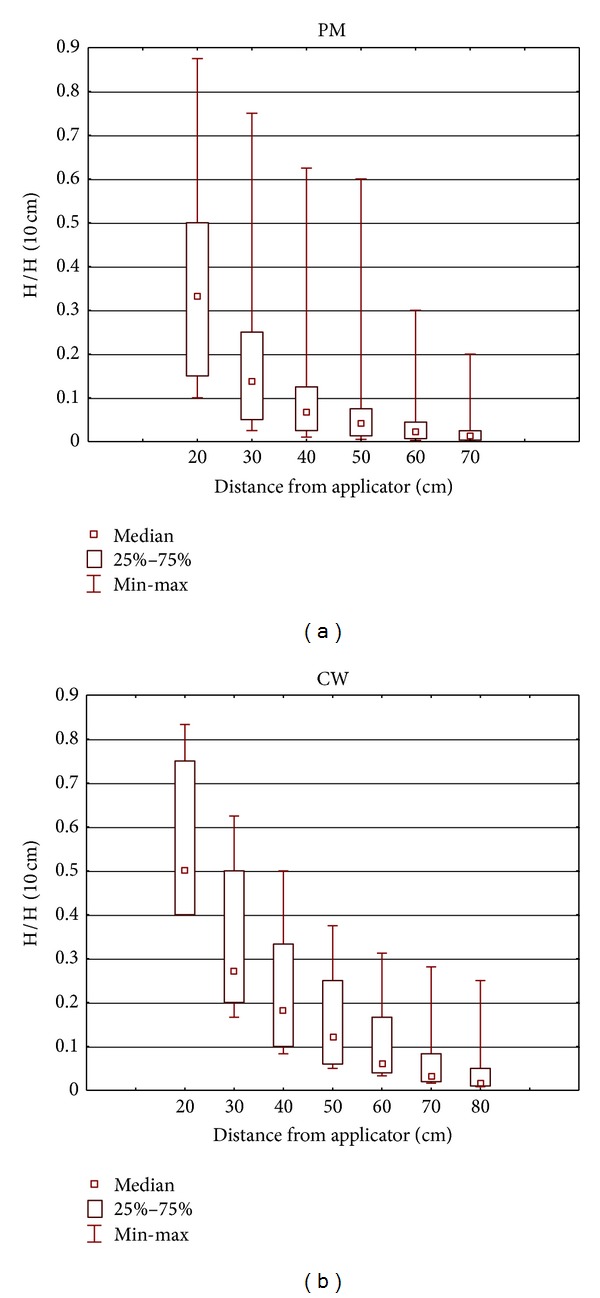
The normalised distribution of the H-field (to the H-field measured at a distance of 10 cm from the cover of applicator) near the applicators. The statistical parameters of all measurement results: (a) SWD-H emitting PM EMF (no. = 9); (b) SWD-E emitting CW EMF (no. = 3).

Each applicator is characterised by a different distribution of the emitted EMF, but for the purpose of identifying the hazards that may be caused by interaction with the human body or AIMD, it is important to understand the basic properties of this distribution. The spatial distribution of EMF emitted by the SWD applicators is nonuniform: with increasing distance, the level of EMF decreases significantly. The normalised (to the E- or H-field measured at the 10 cm distance from the cover of applicator) distribution of the E-field and H-field near the applicators is characterised in Figures 3 and 4 by the statistical parameters of all measurement results. In this way, the general characteristic of the EMF distribution is presented. 

The results of measurements performed near each SWD applicator were used to estimate the distance from the applicator where the E-field and H-field exceed particular exposure limitations referred in Section 2 (e.g., 61 V/m 6-minute averaged RMS value (TA) or 28 V/m nonaveraged in-time value (MT)). In this assessment, the measurement results were counted including the measurement uncertainty (~25% in the case of time averaged RMS value analysis directly following the measurement results but ~50% in the case of non-averaged RMS values over the pulses of PM EMF, derived from the RMS measurement results and duty cycle analysis).

**Figure 5:**
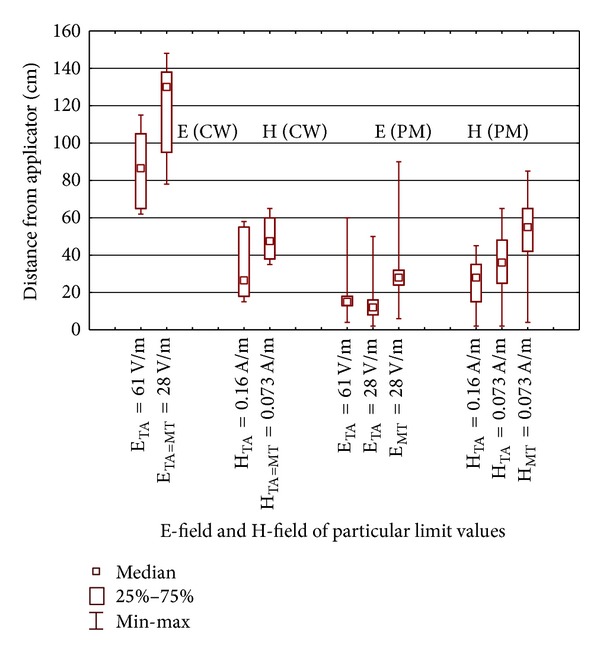
Distance from the SWD applicators, where continues wave (CW) or pulsed modulated (PM) E-field or H-field does not exceed particular exposure limits regarding general public, workers, or AIMD users; E_TA_, H_TA_: electric or magnetic field strength RMS value time averaged over 6 minute; E_MT_, H_MT_: electric or magnetic field strength maximum in-time RMS value.

The level of the E-field exceeding the ICNIRP's reference level on occupational exposure (61 V/m and 0.16 A/m @ 27 MHz, 6 min averaged RMS value) was found at a following distances: E-field at a distance up to 10 cm and H-field up to 45/28 cm (max/median values resp.)—for inductive applicators (SWD-H); but E-field at a distance up to 120/87 cm and H-field up to 58/27 cm—for the capacitive applicators (SWD-E), (Figure 5). 

The distance from the EMF source where the specific exposure limit is fulfilled may be called the “safety distance”—in the case of assessing the workers' exposure limits, the distances mentioned previously may be taken as the “safety distances for workers' exposure.” When workers are present at a closer distance from the active applicator or when the human body is in contact with the EMF source, the previously-mentioned induced electric field and capacitive currents in the body need assessing, which involves numerical calculations or measurements of limb currents [20, 21].

The area where people who not undergoing treatment should not be present (where the ICNIRP's reference levels on general public exposure exceeded-E-field >28 V/m and H-field >0.073 A/m at 27 MHz, 6 min averaged RMS value) can reach a distance of up to 50/12 cm (with regard to the E-field) and 62/38 cm (with regard to the H-field) from the inductive applicators of SWD-H. In the case of capacitive applicators of SWD-E, the EMF of levels exceeding the ICNIRP's general public reference level may be found up to 150/130 cm (with regard to the E-field) and 65/48 cm (with regard to the H-field) from the applicators.

In the assessment of safety of AIMD users, it is necessary to follow modified rules—the limit is based on the nonaveraged in-time RMS value of E- and H-field strengths (28 V/m and 0.073 A/m at 27 MHz, RMS values nonaveraged in-time). As a result, for the continuous wave EMF emitted by the capacitive applicators of SWD-E-the “safety distance for AIMD users” from the applicators is the same as previously-mentioned distance where EMF are compliant with the general public ICNIRP's exposure limitations. But in the case of PM EMF emitted by inductive applicators of SWD-H, the safety distance for AIMD users is significantly longer than the one regarding the exposure for the general public, because the correction factor *K* and elevated uncertainty have to be counted (following the formula 1): a distance of up to 90/28 cm (with regard to the E-field) and 85/55 cm (with regard to the H-field) from the inductive applicators of SWD. 

With respect to the provisions of the new European EMF directive 2013/35/EU regarding the protection of workers' safety and health against direct and indirect hazards near EMF sources, in the process of health surveillance, which should be provided by employers to all workers, the indirect hazards related to EMF emitted by SWD should be managed for workers using AIMD. Informing about such hazards is also an important element of worker information on workplace safety issues. Information on the possible hazards for the AIMD users is also important for every patient and their attendants visiting health care centres, where SWDs are in use.

## 4. Conclusions

Direct and indirect electromagnetic hazards were found near SWD devices in the distance from applicators not exceeding 150 cm—similar to previous reports from the literature [12, 14, 15]. Direct EMF hazards for humans near inductive applicators were identified at a distance not exceeding 45 cm (W) or 62 cm (GP), but for AIMD users up to 90 cm (twice longer than that for W and 50% longer than that for GP—because EMF is pulsed modulated). Near capacitive applicators emitting continuous wave it is 120 cm (W) or 150 cm (GP or AIMD users). The exposure of physiotherapists and other workers present in the therapeutic centre to EMF depends on the workspace spatial organisation and their location while treating patients.

The results of the investigations indicate that there is a need for organisational measures to protect workers against excessive exposure to EMF (e.g., the position of the worker not closer than 50 cm from the SWD applicators while treating patients; the adjustment of the location of applicators by a patient only when EMF is switched off). 

Workers and other AIMD users should be aware that their presence closer than 150 cm from the SWD is not recommended because of a possible implant dysfunction. Warning signs are also recommended to indicate such hazards with respect to the privacy of interested AIMD users [16, 22]. The location of SWD in the electromagnetically shielded boxes may be also recommended to reduce the volume of the space affected by EMF near the active SWD.

In the EU directive 2013/35/EU it is advised the individual analysis of EMF hazards for each AIMD user because of many factors influencing the results of EMF exposure, like the type and model of AIMD, the settings of its operation, the duration and spatial distribution of exposure of the user, and so forth. In the case of well-documented status of AIMD of particular user, it may be achievable for the employer to get supporting data from the AIMD manufacturer. In the result of the technology progress, many modern AIMDs from leading manufacturers are less sensitive to EMF interaction than EN 50527-1:2010 indications, but AIMDs of various designs including old models are still in use, which cause very big variations of the sensitivity level [23]. In general case, individual data for EMF hazards assessment may not be achievable, especially regarding the persons who are not permanently employed in the physiotherapy centre (including patients and attendants). Because of that, for the employer and for a body in charge of the safety of visitors in the physiotherapy centre, it is important to present in this paper general data related to the direct and indirect EMF hazards for workers, patients, and other persons present near active SWD, including AIMD users safety. It is also important to update the safety data, because SWDs and AIMDs design are changing, and becouse the range of EMF which need supervision near SWD may significantly changed over decades. Because of that aspect, further extensive investigations of presented aspect of environmental EMF are suggested.

## Figures and Tables

**Table 1:** The main parameters of investigated short-wave diathermia (SWD).

SWD type	Technical parameters of SWD			Settings of technical parameters during measurements	
	The type of applicator	The averaged output power regulation range (W)	Duty cycle regulation range, *D*	Averaged output power (W)	Duty cycle, *D*
Phyaction Performa+	Thermoplode, Ø 14 cm/H	4.5–90	0.0016–0.45	90	0.45

Curapuls 419	Circuplode, Ø 14 cm/H	6.3–84	0.0063–0.084	84	0.084

Thermo 500	Thermoplode, Ø 14 cm/H	5–70	0.0016–0.35	70	0.35

Curapuls 670	Circuplode-E, 30 × 10 cm/H	0.34–32	0.0017–0.16	32	0.16

Curapuls 670	Circuplode, Ø 9 cm/H	0.17–16	0.0017–0.16	16	0.16

Curapuls 670	Circuplode, Ø 9 cm/H	0.34–32	0.0017–0.16	32	0.16

Terapuls GS 200	Ø 20 cm/H	4.8–60	0.0048–0.06	60	0.06

Phyaction Performa+	Thermoplode, Ø 8 cm/H	3.2–32	0.0016–0.32	32	0.32

Thermatur 500	Circular plates, Ø 16 cm/E	9–90	Continuous wave (CW)	80	CW

Cosmogamma SW500	Circular plates, Ø 14 cm/E	0–500	Continuous wave (CW)	500	CW

Thermopulse	Circular plates, Ø 15 cm/E	10–150	Continuous wave (CW)	150	CW

The type of applicators: H: inductive, E: capacitive.
